# Supplementary material for: A UMLS-based spell checker for natural language processing in vaccine safety
Source: BMC Med Inform Decis Mak. 2007 Feb 12;7:3. doi: 10.1186/1472-6947-7-3 (PMC1805499; doi:10.1186/1472-6947-7-3)
Supplement: Additional file 13 — RAP application directory. Sets up directory for RAP (RDF application for PHP) source code [file 1472-6947-7-3-S13.gz › rap/doc/phpdoc/ontModel/OntVocabulary.html]

xml version="1.0" encoding="iso-8859-1"?


Docs For Class OntVocabulary


## Class OntVocabulary

Description

Description |
Descendents

Base Class that encapsulates the elements of a general vocabulary

corresponding to a particular ontology language. The intent is that, using a given vocabulary, a given RDF model can be processed as an ontology description, without binding knowledge of the vocabulary into this Class

History: <LI>10-07-2004 : First version of this class.</LI>

- access: public
- author: Daniel Westphal <at d-westphal dot de mail at d-westphal dot de>
- version: V0.9.1

Located in /ontModel/OntVocabulary.php (line 27)

Direct descendents

Description |
Descendents

| Class | Description |
| --- | --- |
| OWLVocabulary | OWL vocabulary items |
| RdfsVocabulary | RDFS vocabulary items |

Documentation generated on Thu, 7 Jul 2005 13:42:34 +0200 by phpDocumentor 1.3.0RC3
